# Clinical value of perivascular fat attenuation index and computed tomography derived fractional flow reserve in identification of culprit lesion of subsequent acute coronary syndrome

**DOI:** 10.3389/fcvm.2023.1090397

**Published:** 2023-06-02

**Authors:** Minggang Huang, Tingting Han, Xuan Nie, Shunming Zhu, Di Yang, Yue Mu, Yan Zhang

**Affiliations:** Shaanxi Provincial People’s Hospital, Xi'an, China

**Keywords:** coronary computed tomography angiography, acute coronary syndrome, fat attenuation index, fraction flow reserve, plaque characteristics

## Abstract

**Purpose:**

To explore the potential of perivascular fat attenuation index (FAI) and coronary computed tomography angiography (CCTA) derived fractional flow reserve (CT-FFR) in the identification of culprit lesion leading to subsequent acute coronary syndrome (ACS).

**Methods:**

Thirty patients with documented ACS event who underwent invasive coronary angiography (ICA) from February 2019 to February 2021 and had received CCTA in the previous 6 months were collected retrospectively. 40 patients with stable angina pectoris (SAP) were matched as control group according to sex, age and risk factors. The study population has a mean age of 59.3 ± 12.3 years, with a male prevalence of 81.4%. The plaque characteristics, perivascular fat attenuation index (FAI), and coronary computed tomography angiography-derived fractional flow reserve (CT-FFR) of 32 culprit lesions and 30 non-culprit lesions in ACS patients and 40 highest-grade stenosis lesions in SAP patients were statistically analyzed.

**Results:**

FAI around culprit lesions was increased significantly (−72.4 ± 3.2 HU vs. −79.0 ± 7.7 HU, vs. −80.4 ± 7.0HU, all *p* < 0.001) and CT-FFR was decreased for culprit lesions of ACS patients [0.7(0.1) vs. 0.8(0.1), vs.0.8(0.1), *p *< 0.001] compared to other lesions. According to multivariate analysis, diameter stenosis (DS), FAI, and CT-FFR were significant predictors for identification of the culprit lesion. The integration model of DS, FAI, and CT-FFR showed the significantly highest area under the curve (AUC) of 0.917, compared with other single predictors (all *p* < 0.05).

**Conclusions:**

This study proposes a novel integrated prediction model of DS, FAI, and CT-FFR that enhances the diagnostic accuracy of traditional CCTA for identifying culprit lesions that trigger ACS. Furthermore, this model also provides improved risk stratification for patients and offers valuable insights for predicting future cardiovascular events.

## Introduction

Coronary computed tomography angiography (CCTA) is a first-line imaging method to exclude obstructive coronary artery disease (CAD) and characterize the high-risk plaque features (1, 2). Vascular inflammation is a driving factor of atherosclerotic plaque progression and high-risk plaque rupture might lead to acute coronary syndrome(ACS) (3). ACS (Acute Coronary Syndrome) is a collection of clinical syndromes resulting from the rupture or erosion of coronary atherosclerotic plaques, which leads to partial or complete occlusive thrombosis of the coronary arteries. The three main types of ACS are ST elevation myocardial infarction (STEMI), non-ST elevation myocardial infarction (NSTEMI), and unstable angina (UA). Recent studies revealed that there was an interaction mechanism between the perivascular adipose tissue (PCAT) and vascular wall (4). When adipose tissue was dysfunctional, it would release a large number of pro-inflammatory cytokines through endocrine or paracrine pathways, which would affect the development and destabilization of plaque. Therefore, the perivascular fat attenuation index (FAI) as a new imaging biomarker could reflect coronary inflammatory activity using quantification of adipose tissue attenuation gradient of conventional CCTA. CRISP-CT studies showed perivascular FAI improved the ability of cardiac risk prediction and re-stratification of traditional CCTA, the higher the perivascular FAI value [cutoff ≥ −70.1 Hounsfield Units, (HU)], the higher the risk of cardiogenic mortality and poor prognosis, which suggested that the change of perivascular fat attenuation was closely related to plaque stability (5).

Meanwhile, CTA-derived fractional flow reserve(CT-FFR) is developed as a non-invasive hemodynamic assessment of coronary artery stenosis. Several clinical trials confirmed that CT-FFR could be used to identify lesion specific ischemia, and with good diagnostic performance compared with the gold standard invasive FFR (6–9). Moreover, studies revealed that the integration of CT-FFR and conventional CCTA could greatly improve the identification of culprit lesions that subsequently lead to ACS (10–12).

It is worth noting that plaque rupture is a complex biomechanical process affected by the plaque anatomical structure and composition, as well as the external mechanical and hemodynamic forces acting on the plaque (13). However, the traditional CCTA based on anatomical stenosis assessment lacks functional evaluation of lesions and cannot accurately guide clinical decision-making, resulting in delayed treatment and even subsequent major adverse cardiovascular events (MACE) in some patients. Therefore, this study was conducted to assess the value of indicators derived from CCTA (FAI and CT-FFR) and their integration in the identification of culprit lesions that caused subsequent ACS.

## Materials and methods

### Study population

**Figure 1:**
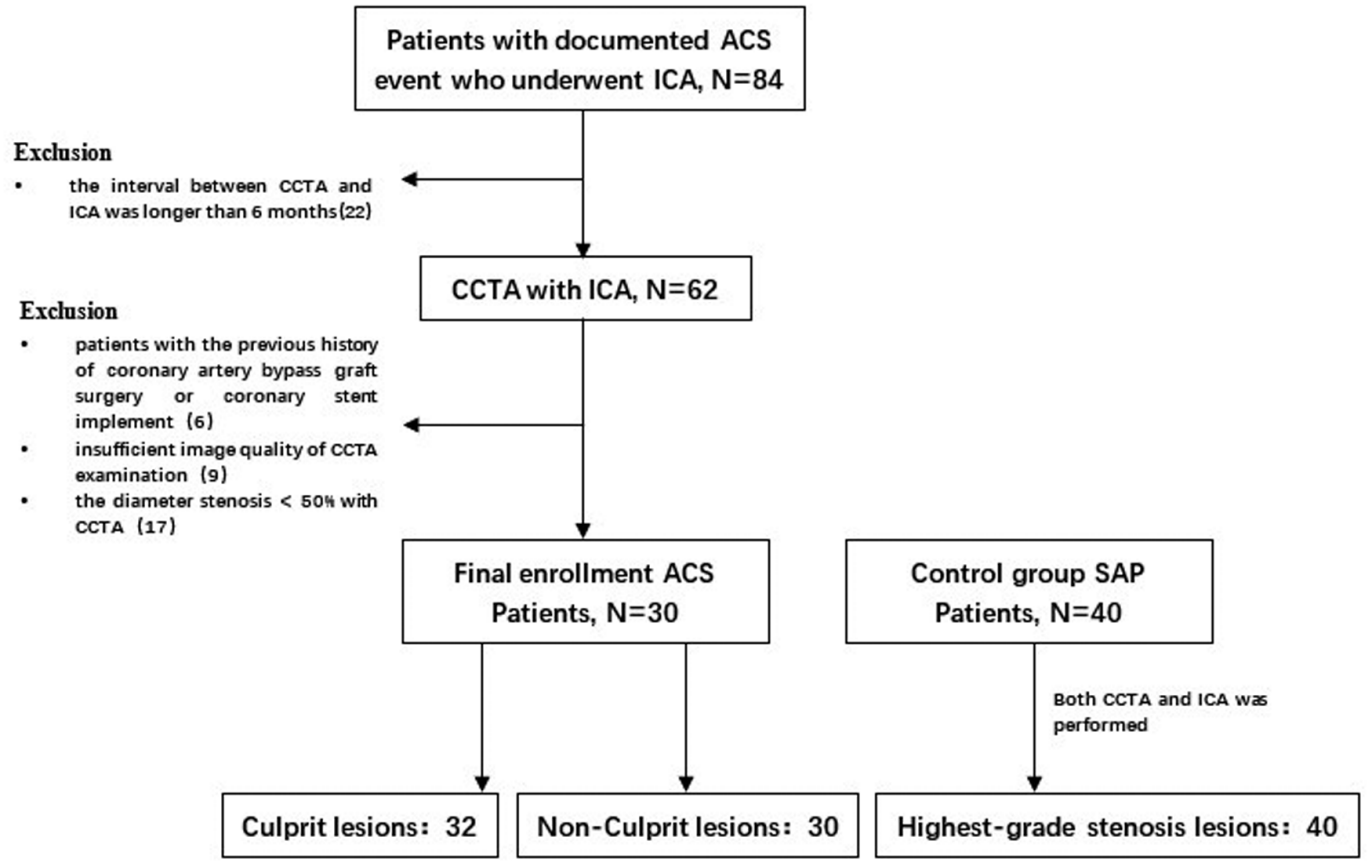
Study flow chart. After screening of enrollment criteria and image quality, a total of 102 lesions in 30 patients with ACS and 40 patients with SAP in the control group were analyzed. ACS, acute coronary syndrome; SAP, stable angina pectoris; ICA, invasive coronary angiography; CCTA, coronary computed tomography angiography.

Thirty patients with documented ACS event who underwent ICA from February 2019 to February 2021 and received at least once CCTA in the previous 6 months were collected retrospectively. Compared with 40 patients with SAP were matched as control group according to sex, age and risk factors, the controls also underwent coronary CTA followed by invasive angiography. The inclusion criterion as follows:(i) the interval between the CCTA examination and ICA within 6 months; (ii) patients with imaging evidence of CAD; (iii) Electrocardiogram (ECG) or Cardiac biomarkers showed no abnormalities. The exclusion criteria were as follows: (i) the interval between CCTA and ICA was longer than 6 months; and ICA was longer than 6 months; (ii) patients with the previous history of coronary artery bypass graft surgery or coronary stent implement; (iii) insufficient image quality of CCTA examination; (iv)The diameter stenosis <50% with CCTA (Figure 1. Study Flow Chart). The study was in accordance with the regulations of the institutional review committee.

### CCTA acquisition

All patients underwent CT imaging with a 320-row wide area detector CT scanner (Acquilion ONE VISION Edition). Prospective or retrospective ECG-triggered sequential acquisition according to the patient's heart rate were applied. The detailed scanning parameters were as follows: the scanning range was from the tracheal bifurcation level to the diaphragmatic surface of the heart, and the cross-section of the aortic root was set as the monitoring section. The trigger scan was carried out by using Sure Start contrast medium tracking software, and the threshold was 300∼330HU; collimation was 320 × 0.5 mm;100 kv tube voltage; tube current modulated with automatic exposure control (AEC) technology with noise index (SD = 26); rotation speed 0.275 rot/s; images were reconstructed with AIDR3D with standard kernel (FC = 09), thickness was 0.5 mm with interval 0.25 mm. Two-barrel high-pressure syringe was used to inject 60 ml nonionic contrast agent iopromide (370 mg/ml) or iodixanol (320 mg/ml) and 30 ml normal saline through the right elbow vein at 4.5∼5.5 ml/s flow rate.

### Plaque characteristics

The images with the good image quality were selected for further postprocessing and plaque analysis (Vitrea Advanced FX3.0, Canon Medical Systems). The plaques'characterization included: (1) remodeling index (RI) defined as the ratio of the cross-sectional vessel lesion area to the proximal reference vessel area; positive reconstruction (PR) was defined as a RI > 1.0; (2) the diameter stenosis (DS) calculated as: (reference diameter-minimal lumen diameter)/reference diameter; (3) total plaque (TP) burden (PB%) calculated as follows: PB = plaque volume × 100%/vessel volume; (4) calcified plaque [CP, 376 to 1300(HU)] burden; (5) non-calcified burden [NCP, −30 to 375(HU)] burden. As previously described (14), NCP was further divided into three types: low-attenuation NCP [L-NCP, −30 to 30(HU)], intermediate-attenuation [I-NCP, 31 to 130(HU)], and high-attenuation NCP [H-NCP, 131 to 375(HU)] volumes, and their corresponding plaque burden.

### Perivascular FAI

FAI was defined as the average CT attenuation of perivascular adipose tissue within a radial distance from the outer vessel wall equal to the diameter of the target vessel (4, 5). A dedicated FAI analysis software (EasyFAI Intelligent Evaluation system of perivascular Fat, version 1.2.0, Shukun, China) was used for quantification. As described previously (4), the average CT attenuation between −190HU and −30HU of perivascular adipose tissue with the edge of vascular wall extending outward by 4 mm was measured. The longitudinal range was 40 mm from the distal end of left anterior descending branch (LAD) and left circumflex branch (LCX). To avoid the effects of the aortic wall, we excluded the most proximal 10 mm of the right coronary artery (RCA) and analyzed the proximal 10 mm to 50 mm of the vessel.

### CT-FFR

An artificial intelligent based CT-FFR software DEEPVESSEL (KEYA Medical, China), which was approved for commerical use by Chinese National Medical Products Administration (NMPA), was used to calculate the CT-FFR values. The software was fully valdidated with multiple clinical trials and the reported accuracy ranged from 87.3% to 90.4% using the invasive FFR as the reference (15, 16). The software utilize deep learning alogirhtms for CT-FFR calculation, which uses an advanced neural network architecture that was trained offline to learn the complex mapping between the embedded anatomatical and lesion features and its corresponding hemodynamics from the coronary artery tree. It takes the centerline and images as inputs and calculates CT-FFR for the whole artery tree. The model fully considers the artery tree structure, lesion-specific information, spatial relationship, and the influences of other branches for the blood flow dynamics. Please refer to (17) for detailed descriptions. The CT-FFR value at the distal 2–3 cm of the stenosis was recorded.

### Statistical analysis

Statistical analysis was performed using SPSS (version 25.0, IBM Corp., Armonk,N.Y., USA, and MedCalc Statistical Software). Shapiro-Wilk test was used to check the assumption of normal distribution. Quantitative variables with normal distribution were expressed as mean ± standard deviation (SD) while median and quartile spacing were used for variables that were not normally distributed. T-test and Pearson test were used for data with normal distribution while Mann–Whitney U-test was used otherwise. Categorical variables were reported as count (%). To determine the optimal model for identifying culprit lesions, a multivariable logistic regression analysis was conducted with the “enter” approach. The model consisted of variables with *p* < 0.10 in the univariate analysis. Variables that were statistically significant in univariate analysis were included for further receiver operating characteristic (ROC) curve analysis. The optimal cutoff values for various parameters were determined using the maximum sum of sensitivity and specificity in ROC curve analysis. The area under the curve (AUC) was compared using the DeLong method (18). A two-tail probability value of *p* < 0.05 was considered statistically significant.

## Results

### Clinical characteristics

The clinical characteristics of the study population were listed in Table 1. In total 70 patients with 102 lesions (30 patients with ACS, including 9 patients with non-ST segment elevation myocardial infarction, 6 patients with ST segment elevation myocardial infarction, 15 patients with unstable angina pectoris, and 40 patients with SAP) were finally included (mean age: 59.3 ± 12.3, 81.4% of males and an average BMI of 23.8 ± 2.7 kg/m^2^). There were no significant differences in age, sex, BMI, coronary risk factors, laboratory indexes between patients with ACS and SAP (all *p* > 0.05). Since there were patients with non-ST segment elevation acute coronary syndrome with multivessel coronary artery disease in the study cohort, we included 32 culprit lesions and 30 non-culprit lesions with less stenosis than culprit lesions in patients with ACS. 40 highest-grade stenosis lesions in SAP patients were further analyzed. The culprit lesion often arise from non-obstructive plaque due to acute plaque rupture, and were defined as that subsequently lead to acute coronary syndrome in this study. To identify the potential culprit lesion site, information derived from angiographic images and ECGs was integrated as per common clinical practice. Culprit coronary lesions were determined on the angiographic findings suggestive of plaque rupture according to ESC guidelines for the management of acute coronary syndrome patients (19).

**Table 1:** Clinical characteristics.

Characteristics	Patients wth ACS (30)	Patients with SAP (40)	t/x^2^	*P*-Value
Demographics				
Ages (years)^b^	56.3 ± 10.6	61.5 ± 13.1	1.802	0.076
Male, *n* (%)	22 (73%)	35 (88%)	2.275	0.131
BMI (kg/m2)^b^	24.0 ± 2.2	23.6 ± 3.0	0.584	0.561
Cardiovascular risk factors, *n* (%)				
Diabetes	14 (47%)	17 (43%)	0.121	0.728
Hypertension	17 (57%)	25 (63%)	0.243	0.622
Hypercholesterolemia	14 (47%)	13 (33%)	1.452	0.228
Current smoker	18 (60%)	20 (50%)	0.691	0.406
Drinking history	9 (30%)	8 (20%)	0.932	0.334
Family history	8 (27%)	7 (18%)	0.856	0.355
Lipid markers(mmol/l)				
TC^b^	4.6 ± 1.1	4.4 ± 0.8	−0.962	0.340
TG^a^	1.7 (1.45)	1.3 (0.86)	0.831	0.406
LDL^b^	2.7 ± 0.7	2.6 ± 0.6	0.314	0.754
HDL^a^	1.2 (0.21)	1.2 (0.25)	0.623	0.533

ACS, acute coronary syndrome; SAP, stable angina pectoris; BMI, body mass index; TC, total cholesterol; TG, triglyceride; LDL, low density lipoprotein; HDL, high density lipoprotein.

^a^ Data are medians, with quartile spacing in parentheses.

^b^ Data are mean ± the standard deviation (SD).

### Plaque characteristics

Table 2 showed the comparison of plaque characteristics in different lesion types. The burden of TP, L-NCP and I-NCP were significantly increased in culprit lesions compared with non-culprit lesions in patients with ACS (66.5% vs. 57.7%, 12.3% vs. 11. 1%, 35.7% vs. 30.2%, all *p *< 0.05). (Table 2) The burden of TP, NCP and TP volume were significantly increased in culprit lesions compared with highest-grade stenosis lesions in patients with SAP (66.5% vs. 58.9%, 63.4% vs. 57.3%, 237.0 mm^3^ vs. 178.0 mm^3^, all *P *< 0.05). It is worth noting that the culprit lesions with ACS had higher DS, and RI, and longer lesion length compared with other lesions. (Table 2) In addition, perivascular FAI of culprit lesions was significantly higher than the non-culprit lesions of patients with ACS and the highest-grade stenosis lesions in SAP patients (−72.4 ± 3.2 vs. −79.0 ± 7.7, vs. −80.4 ± 7.0, *p *< 0.001), while the CT-FFR of culprit lesions was significantly lower than that of other two groups[0.7(0.1) vs.0.8(0.1), vs.0.8(0.1), *p *< 0.001].

**Table 2:** Plaque characteristics of culprit lesion and non-culprit lesion in patients with ACS and the highest-grade stenosis in patients with SAP.

	Patients with ACS		*P*-Value	Patients with SAP	*P*-Value
	Culprit Lesions	Non-culprit		Highest-grade	
Plaque burden (%)					
TP	66.5 ± 12.1	57.7 ± 9.1	0.002^b^	58.9 ± 8.9	0.003^a^
CP^a^	0.2 (2.2)	0.01 (2.0)	0.615	0.0 (1.3)	0.167
NCP	63.4 ± 10.5	55.1 ± 9.5	0.002^b^	57.3 ± 9.3	0.011^b^
Low-attenuation					
NCP^a^	12.3 (4.3)	11.1 (4.2)	0.031^b^	11.3 (3.2)	0.158
Intermediate-attenuation					
NCP^a^	35.7 (9.9)	30.2 (13.8)	0.021^b^	33.9 (14.2)	0.127
High-attenuation					
NCP^a^	12.2 (11.0)	14.3 (12.7)	0.535	10.9 (11.3)	0.932
Plaque volume, mm3					
TP^a^	237.0 (151.0)	201.0 (154.0)	0.205	178.0 (136.0)	0.038^b^
Low-attenuation					
NCP^a^	44.4 (32.9)	36.8 (15.5)	0.086	39.6 (22.3)	0.057
Intermediate-attenuation					
NCP^a^	128.2 (94.9)	106.8 (72.0)	0.065	105.1 (72.2)	0.055
High-attenuation					
NCP^a^	45.7 (41.2)	46.2 (50.5)	0.871	32.7 (32.7)	0.111
Plaque Characteristics					
DS, %	82.5 (33.0)	48.5 (28.0)	<0.001^b^	67.0 (44.0)	0.089
RI^a^	1.3 (0.4)	1.1 (0.4)	0.017^b^	1.0 (0.3)	<0.001^b^
Lesion length (mm)^a^	25.2 (13.7)	23.1 (7.1)	0.278	20.6(7.7)	0.017^b^

TP, total plaque; CP, calcified plaque; NCP, non-calcified; RI, remodeling index; DS, diameter stenosis.

^a^ Data are medians, with quartile spacing in parentheses.

^b^ indicated statistical significance.

### Identification of culprit lesions that caused subsequent ACS

In univariate analysis, perivascular FAI, CT-FFR, RI, DS, and the burden of TP, NCP, L-NCP and I-NCP were significantly related to the presence of culprit lesions (*p* < 0.05 for all). Further multivariate logistic regression analysis showed that DS, CT- FFR, and perivascular FAI were still independent predictors of culprit lesions (all *p* < 0.05) (Table 3).

**Table 3:** Univariate and multivariate analyses of culprit lesions.

	Univariate analysis		Multivariate analysis	
	0R (95%CI)	*P*-Value	0R (95%CI)	*P*-Value
TP	1.088 (1.025–1.155)	0.006^a^	0.958 (0.797–1.153)	0.651
NCP	1.092 (1.027–1.161)	0.005^a^	1.107 (0.831–1.475)	0.486
Low-attenuation				
NCP	1.265 (1.047–1.530)	0.015^a^	1.297 (0.832–2.020)	0.251
Intermediate-				
attenuation NCP	1.085 (1.019–1.155)	0.011^a^	0.897 (0.740–1.089)	0.272
RI	1.027 (1.005–1.050)	0.018^a^	1.067 (0.992–1.148)	0.081
DS	1.080 (1.041–1.121)	<0.001^a^	1.093 (1.000–1.195)	0.049^a^
FAI	1.242 (1.097–1.406)	0.001^a^	1.309 (1.005–1.705)	0.046^a^
CT-FFR	0.711 (0.600–0.842)	<0.001^a^	0.638 (0.430–0.946)	0.025^a^

TP, total plaque; NCP, non-calcified; RI, remodeling index; DS, diameter stenosis; FAI, fat attenuation index; FFR, fractional flow reserve; OR, odds ratio.

^a^ indicated statistical significance.

### Predictive value of markers for the identification of ACS

**Figure 2:**
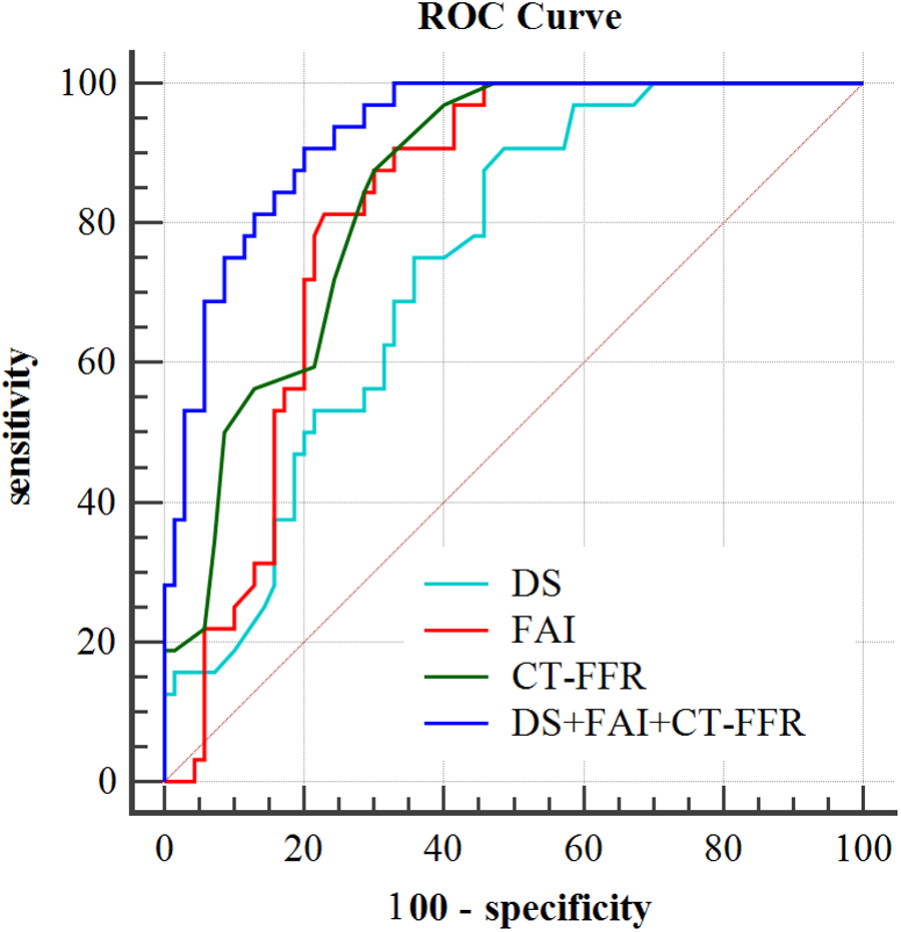
Incremental value of single factor and integrated model detection for ACS.

According to ROC curve analysis, the AUC of DS in culprit lesions was 0.72 (95%CI, 0.623–0.817), with the best cut-off value of 54.5%, a sensitivity of 90.6% and a specificity of 51.4%. The AUC of FAI in culprit lesions was 0.817(95%CI: 0.737–0.898), with the best cut-off value of −74.8HU, a sensitivity of 81.3% and a specificity of 77.1%. For single parameters, CT-FFR showed the highest AUC (AUC = 0.849, 95%CI: 0.778–0.921) for identification of culprit lesions of subsequent ACS, with the best cut-off value of 0.795, a sensitivity of 87.5% and a specificity of 70% (Table 4). The frequency of CT-FFR ≤ 0.795 in culprit lesions was higher than that of non-culprit lesions (87.5% vs. 30%). When incoprating DS, CT-FFR, and perivascular FAI, the AUC of the combined model increased to 0.917, with sensitivity of 96.6%, and specificity of 74.3% (Table 4). In addition, combined approach had was revealed to have the significant larger AUC than any other single parameters for identification of culprit lesions of subsequent ACS (both *p* < 0.05) (Figure 2).

**Table 4:** Performance comparison of ROC curve analysis for identifying the culprit lesions leading to subsequent ACS events.

	AUC [95%CI]	Cut-off Value	Sensitivity (%)	Specificity (%)
DS	0. 720 ( 0.623 -0.817 )*	5 4.5	0.906	0.514
F A I	0. 817([ 0. 737 -0.8 98)*	−74.79	0.813	0.771
CT-FFR	0.8 49 ( 0.778 -0.921 )*	0.795	0.875	0.700
F A I + CT -F F R + DS	0.9 17 ( 0.866 -0.968)	−1.2349	0.969	0.743

DS, diameter stenosis; FAI, fat attenuation index; FFR, fractional flow reserve; ROC, receiver operating characteristic. CI, confidence interval.

* *P* < 0.05 vs. FAI + CT-FFR + DS.

## Discussion

This study investigated the ability of comprehensive evaluation using FAI and CT-FFR in the identification of culprit lesions that caused subsequent ACS. The major finding of this study was that perivascular FAI and CT-FFR are independent risk factors for culprit lesions in patients with ACS and the integration model of DS, FAI, and CT-FFR significantly improved the traditional CCTA for identification of culprit lesions that subsequently lead to ACS, with highest AUC.

Previous study found that perivascular FAI changes dynamically with plaque progression in patients with ACS (4, 20). The reason was inflammation of coronary artery can inhibit the formation of perivascular adipose tissue and increase the CT attenuation of local adipose tissue. Therefore, FAI was considered to be a vessel wall thermometer which promoted the non-invasive assessment of coronary artery inflammation phenotype. The results of this study showed that FAI increased around culprit vessels compared with non-culprit vessels, which was consistent with the results of previous studies (14, 21). The vessel wall could release a large number of inflammatory signals through the paracrine pathway to affect the biological processes such as adipocyte differentiation, proliferation, and lipolysis, leading to the local fat-water phase transition, which is finally manifested by the increase of fat attenuation density around the coronary artery in CCTA (22).

CCTA can be used as an ideal imaging method for non-invasive monitoring of the anatomical characteristics of high-risk coronary plaque, specifically positive remodeling and/or low attenuation plaque are occasionally regarded as similar to those of the culprit plaque once an ACS has occurred (23–25). A recent study found that dynamic changes of plaque might be highly related to subsequent ACS events. A machine learning approach considering comprehensive lesion characteristics (e.g., CT-FFR, necrotic core, remodeling index, plaque volume, and calcium) could improve the ability for predicting risks of ACS events (26). In addition, study found that the low attenuation plaques (LAP) volume of thin-cap fibroatheroma (TCFA) lesions quantified by CCTA was significantly higher than that of non-TCFA lesions, and IVUS confirmed that LAP volume was the strongest predictor of TCFA lesions (27). Williams et al. found that low attenuation NCP burden is the main predictor of prognosis, and patients with stable chest pain with low attenuation NCP burden > 4% are 5 times more likely to develop fatal or non-fatal myocardial infarction than the control group (28). In our study, the burden and volume of TP, L-and I- NCP were increased in culprit lesions compared with both non-culprit lesions in patients with ACS and highest-grade stenosis lesions, with a higher degree of stenosis and remodeling index. It was further confirmed that plaque characteristics were more closely related to culprit lesions that subsequently led to the risk of ACS, which was consistent with previous studies (29, 30). Interestingly, Motoyama et al. reported that about 83.7% of high-risk plaques did not cause ACS events (24). The reason is that plaque rupture is an extremely complex biomechanical process, which is not only related to the plaque structure and components, but also the comprehensive influence of external mechanical force and hemodynamic force acting on the plaque. When the internal structural stress of the plaque exceeds the plaque strength, it will rupture (31, 32).

FFR is currently the gold standard for the hemodynamic evaluation of coronary lesions, which limited in clinical application due to its expensive examination cost and invasive examination procedures. It is worth noting that, as a derivative parameter of conventional CCTA, CT-FFR showed better diagnostic performance than CCTA alone. It can add functional information to the traditional CCTA plaque morphological evaluation and realize the early identification of coronary artery stenosis and ischemic lesions. In our study, the CT-FFR value of culprit lesions in ACS patients was significantly lower than that of non-culprit lesions and the highest-grade stenosis of SAP, and the frequency of decreased in culprit lesions CT-FFR (≤0.795) was higher, which was consistent with the results of Lee JM et al. (11). Therefore, we further explore whether the construction of a combined model can improve the diagnostic performance of subsequent ACS risk culprit lesions.

It has been reported that the perivascular FAI of flow-limited lesions was significantly higher than that of non-flow-limiting lesions, and the combined use of FAI, TP volume and DS could predict ischemic coronary stenosis with high diagnostic accuracy (33, 34). In our study, multivariate analysis showed that DS, FAI, and CT-FFR were independent risk factors for culprit lesions. Compared with single variable, the combined application of FAI, CT-FFR and DS can significantly improve the identify culprit lesions in subsequent ACS events. Our results support that the combination of FAI and CT-FFR assessment could improve the ability to identify culprit lesions in subsequent ACS events compared to traditional CCTA, and provide a one-stop non-invasive comprehensive evaluation of coronary morphology and function without any other further examination.

So far, CAD is still the first killer endangering human health, although it has been quite mature in diagnosis, treatment decision-making and prognosis evaluation. However, relying only on clinical evaluation has some limitations, for example, when patients were in low to intermediate chest pain risk category with normal initial cardiac biomarkers and none had evidence of ischemia on initial ECGs and acute myocardial infarction was subsequently diagnosed in 10% of patients (35). Therefore, there is an urgent need for a comprehensive evaluation system integrating plaque stability and non-invasive hemodynamics to guide clinical decision-making and avoid subsequent adverse events. More importantly, with the increasing global health expenditures, this simple and non-invasive technique might help to control medical costs and reduce many unnecessary and expensive invasive procedures.

Despite the above findings, our study had several limitations. First, this was a single-center retrospective observational study, the sample size was relatively small, and there was a certain selection bias, which limited the clinical applicability and reliability of the conclusion to a certain extent. Second, this study observed that the diagnostic performance of the combined model was significantly improved and the sensitivity was reduced, which may be related to the small sample size, large-scale prospective studies would be needed to verify it in the future. Third, there were no statistics on the use of statins in our study, which may affect the relationship between FAI and plaque vulnerability, which should be further investigated.

In conclusion, the perivascular FAI of culprit lesions in ACS patients was significantly increased and CT-FFR decreased compare to other lesions of ACS and SAP. The integration of DS, FAI, and CT-FFR could significantly improve the ability of traditional CCTA to identify the culprit lesions that caused subsequently lead to ACS. It is plausible that AI-based intelligent technology can furnish additional physiological information regarding coronary artery disease, thereby facilitating personalized risk assessment and identification of coronary atherosclerotic heart disease and ACS.

## Data Availability

The original contributions presented in the study are included in the article, further inquiries can be directed to the corresponding author.
